# Supplementary material for: Bidirectional interaction between protocadherin 8 and the transcription factor Dbx1 regulates cerebral cortex development
Source: Development. 2026 Jan 6;153(1):dev205011. doi: 10.1242/dev.205011 (PMC12848574; doi:10.1242/dev.205011)
Supplement: Supplementary information [file develop-153-205011-s1.pdf]

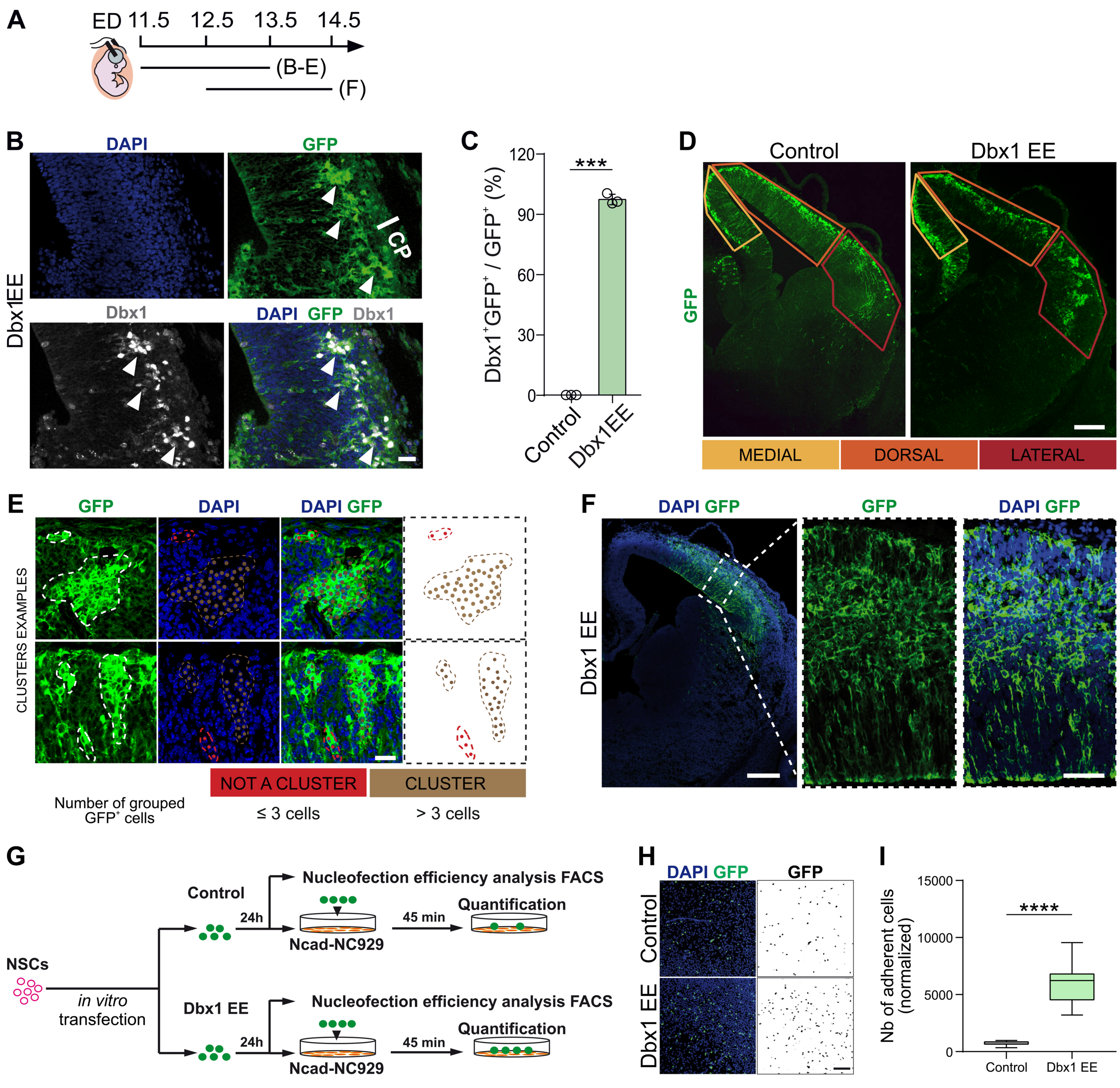
A
ED 11.5 12.5 13.5 14.5
(B-E)
(F)
B
DAPI
GFP
CP
Dbx1EE
Dbx1
DAPI GFP Dbx1
C
Dbx1+GFP+ / GFP+ (%)
120
90
60
30
0
***
Control
Dbx1EE
D
Control
Dbx1 EE
GFP
MEDIAL
DORSAL
LATERAL
E
GFP
DAPI
DAPI GFP
CLUSTERS EXAMPLES
NOT A CLUSTER
CLUSTER
Number of grouped GFP+ cells
≤ 3 cells
> 3 cells
F
DAPI GFP
GFP
DAPI GFP
Dbx1 EE
G
NSCs
in vitro transfection
Control
Dbx1 EE
24h
Nucleofection efficiency analysis FACS
Ncad-NC929
45 min
Quantification
H
DAPI GFP
GFP
Control
Dbx1 EE
I
Nb of adherent cells (normalized)
15000
10000
5000
0
****
Control
Dbx1 EE

Fig. S1. Dbx1 EE at E11.5 induces the formation of cell aggregates in the lateral/ventral pallium through increased adhesiveness.

(A) Timeline of the IUE. ED, electroporation day. **(B)** Confocal images of GFP (green) in coronal sections of E13.5 mouse brains electroporated at E11.5 with Dbx1 EE, co-labeled with Dbx1 (gray) and DAPI (blue) counterstaining. White line shows the C. White arrowheads indicate $Dbx1^{+}GFP^{+}$ cells. Scale bar: 50 µm. **(C)** Percentage of $Dbx1^{+}GFP^{+}$ cells among GFP^{+} cells. Data are mean ± SEM; circles represent values from independent electroporated embryos (n=3 each condition). Student's t-test: ***p<0.001. **(D)** Representative confocal images of GFP (green) in coronal sections of E13.5 mouse brains electroporated at E11.5 with control or Dbx1 EE. The outlined regions indicate the medial, dorsal and lateral pallium masks used for cluster quantification. Scale bar: 200 µm. **(E)** Representative images showing GFP^{+} (green) clusters and non-clustered cells (outlined with dashed lines). Individual GFP^{+} cells (dots) were identified based on nuclear DAPI (blue) staining. A cluster was defined as a group of more than 3 GFP^{+} cells in close proximity. Scale bar: 50 µm. **(F)** Confocal images of GFP (green) in coronal sections of E14.5 mouse brains electroporated at E12.5 with Dbx1 EE, with DAPI (blue) counterstaining. Dashed rectangle magnified on the right. Scale bars: 200, 100 µm (magnified). **(G)** Schematic showing the *in vitro* adhesion assay. Twenty-four hours after transfection, neural stem cells (NSCs) transfected with either a control vector or Dbx1 EE were analyzed by FACS to assess nucleofection efficiency, or seeded onto Ncad-expressing NC929 cell monolayers for 45 min before quantification. **(H)** Confocal images of transfected GFP^{+} (green) cells with a control vector or Dbx1 EE plasmid, with DAPI (blue) counterstaining. Black-and-white images of adherent GFP^{+} cells are show in the right panel. Scale bar: 100 µm. **(I)** Quantification of the number of adherent cells transfected with control or Dbx1 EE vector normalized to the percentage of all GFP^{+} detected by FACS in each corresponding condition. Student's t-test: ****p≤0.0001.

Development • Supplementary information

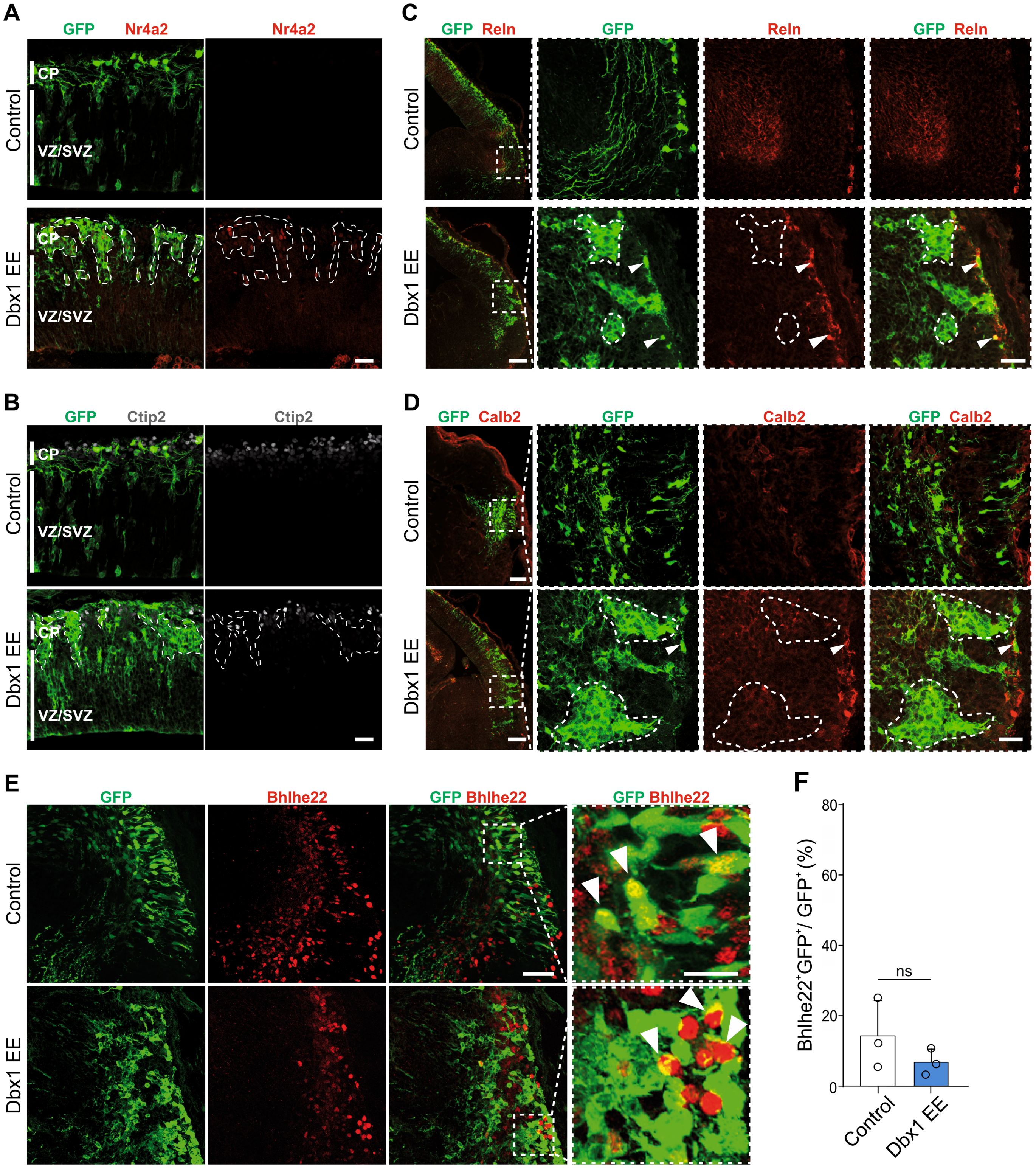

Development • Supplementary information

Fig. S2. Dbx1 EE at E11.5 in the lateral/ventral pallium does not induce a dorsal pallial identity

(A, B) Confocal images of GFP (green) in coronal sections of E13.5 mouse brains electroporated at E11.5 with control vector or Dbx1 EE, co-labeled with **(A)** Nr4a2 (red) and **(B)** Ctip2 (gray). White lines show the CP and the ventricular/subventricular zones (VZ/SVZ). Scale bars: 50 µm. **(C, D)** Confocal images of GFP (green) in coronal sections of E13.5 mouse brains electroporated at E11.5 with control vector or Dbx1 EE, co-labeled with **(C)** Reln (red) or **(D)** Calb2 (red). Scale bars: 200 µm. Dashed squares magnified on the right. Scale bars: 50 µm. White arrowheads indicate **(C)** $GFP^{+}Reln^{+}$ and **(D)** $GFP^{+}Calb2^{+}$ cells localized outside the aggregates. Dashed areas delineate Dbx1 EE-induced aggregates. **(E)** Confocal images of GFP (green) in coronal sections of E13.5 mouse brains electroporated at E11.5 with control or Dbx1 EE, co-labeled with Bhlhe22 (red). Dashed squares magnified on the right. White arrowheads indicate $GFP^{+}Bhlhe22^{+}$ cells. Scale bars: 100 µm; 25 µm (magnified). **(F)** Percentage of $Bhlhe22^{+}GFP^{+}$ cells among GFP^{+} cells. Data are mean ± SEM; circles represent values from independent electroporated embryos (*n*=3 each condition). Student's *t*-test: ns, not significant.

Development • Supplementary information

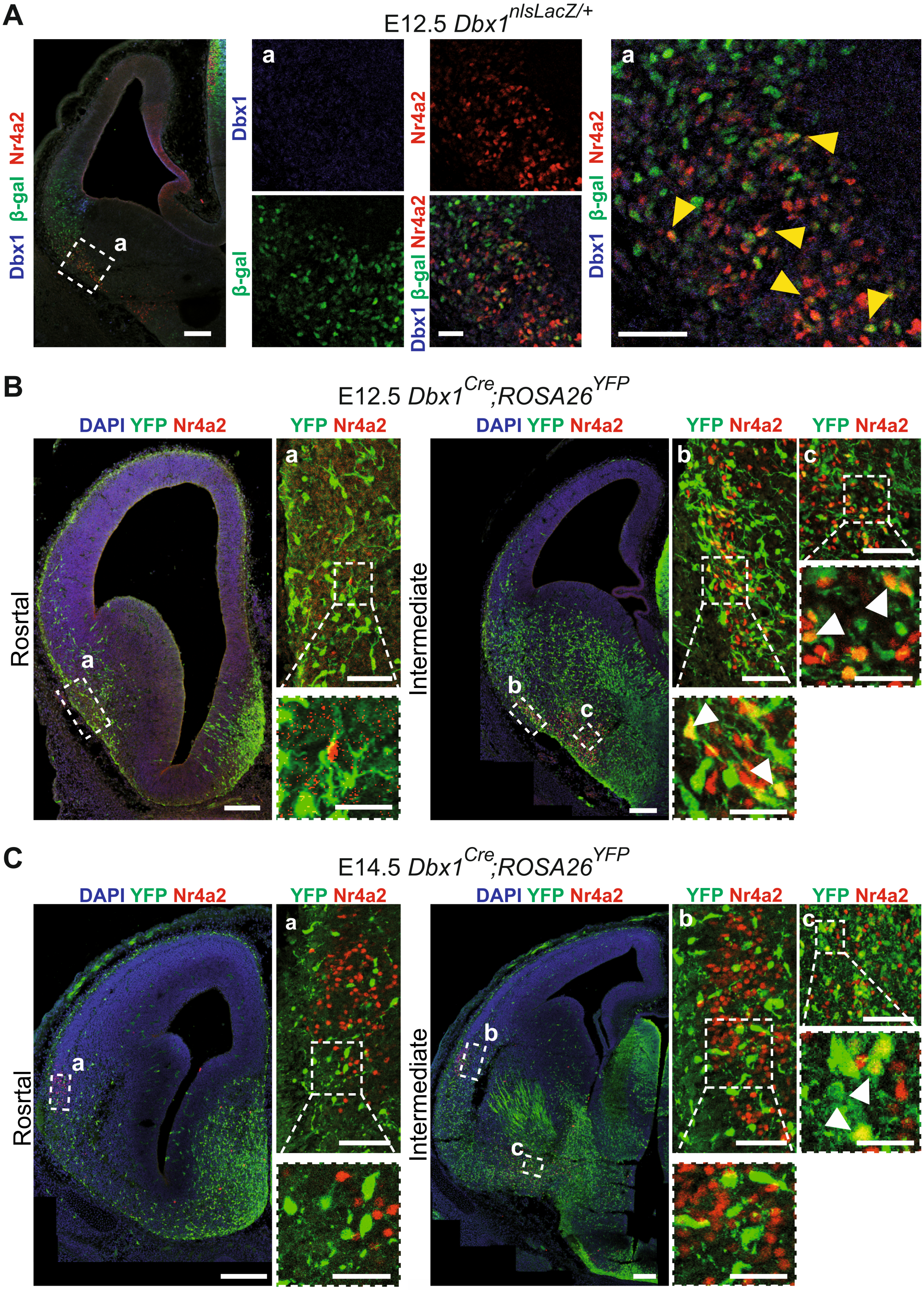
A
E12.5 Dbx1nlsLacZ/+
Dbx1 β-gal Nr4a2
a
Dbx1
Nr4a2
β-gal
Dbx1 β-gal Nr4a2
B
E12.5 Dbx1Cre;ROSA26YFP
DAPI YFP Nr4a2
YFP Nr4a2
Rosrtal
Intermediate
b
c
C
E14.5 Dbx1Cre;ROSA26YFP

Fig. S3. **Dbx1-derived cells express Nr4a2 in the ventral pallium.**

(A) Confocal images of coronal brain sections from E12.5 $Dbx1^{nlsLacZ/+}$ embryos, labeled for β-gal (green), Dbx1 (blue) and Nr4a2 (red). The dashed square (a) magnified on the right. Yellow arrows indicate β-gal^{+}Nr4a2^{+} cells. Scale bars: 100 µm, 50 µm (a). **(B, C)** Confocal images of coronal brain sections from **(B)** E12.5 and **(C)** E14.5 $Dbx1^{Cre}$;$Rosa26^{YFP}$ embryos labeled with YFP (green), Nr4a2 (red) and DAPI (blue). Dashed boxes (a, b, c) magnified on the right of each panel. Dashed squares highlighting selected groups of cells in panels a, b and c magnified below. White arrowheads indicate YFP^{+}Nr4a2^{+} cells. Scale bars: 100 µm; 50 µm (top magnified); 25 µm (bottom magnified).

Development • Supplementary information

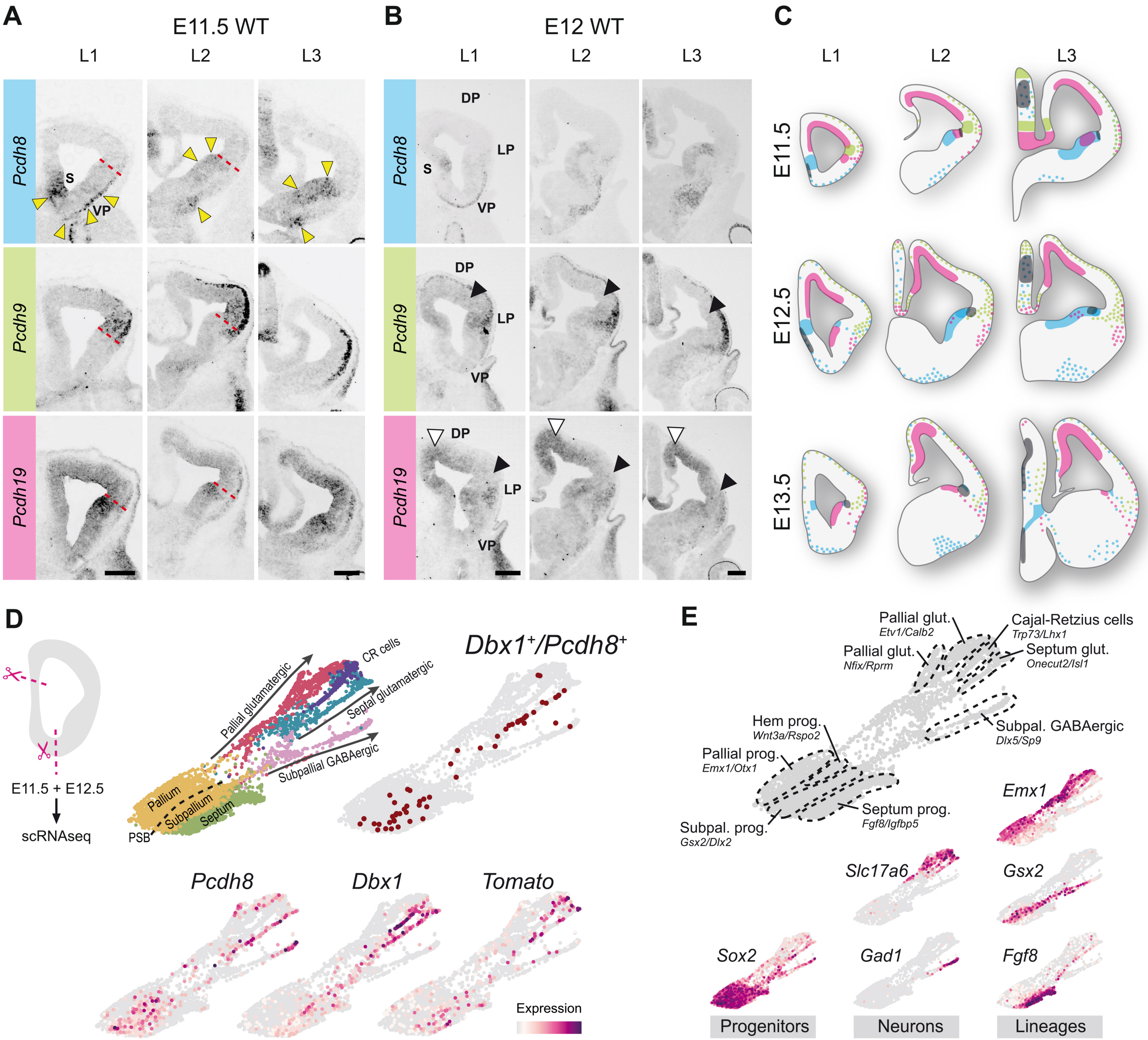

Fig. S4. **δ-Pcdh expression is very dynamic in the embryonic telencephalon and distinct lineage trajectories emerge around the septum.**

(A, B) Bright-field images of in situ hybridization for Pcdh8, Pcdh9 and Pcdh19 in coronal brain sections of (A) E11.5 and (B) E12 WT embryos. Red dashed lines mark the pallial-subpallial boundary. Yellow and white arrowheads indicate regions of high Pcdh8 or Pcdh19 expression, respectively, while black arrowheads indicate progenitor regions with low Pcdh9 and Pcdh19 expression. S, septum; DP, dorsal pallium; LP, lateral pallium; VP, ventral

Development • Supplementary information

pallium. Scale bars: 100 µm. **(C)** Schematic showing *Pcdh* mRNA expression patterns at E11.5, E12.5 and E13.5. The following color code was used: blue for *Pcdh8*, green for *Pcdh9*, pink for *Pcdh19*, grey for *Dbx1*. L1, L2 and L3 correspond to rostral, intermediate and caudal section levels, respectively. **(D)** Top left: Schematic showing the dissection used for scRNAseq profiling. Top: SPRING dimensionality reduction of the septum dataset, with cells colored by cell type and origin. Arrows indicate the main differentiation trajectories, and the dashed line marks the PSB. CR, Cajal-Retzius. Cells co-expressing *Dbx1* and *Pcdh8* are show in dark red (top right). Bottom: expression levels of *Pcdh8*, *Dbx1* and *Tomato* transcripts. **(E)** Top: SPRING representation of the septum dataset showing the distinct progenitor and neuronal populations that were identified, with selected marker genes indicated for each population. Bottom: expression level of selected genes in marked differentiation trajectories.

Development • Supplementary information

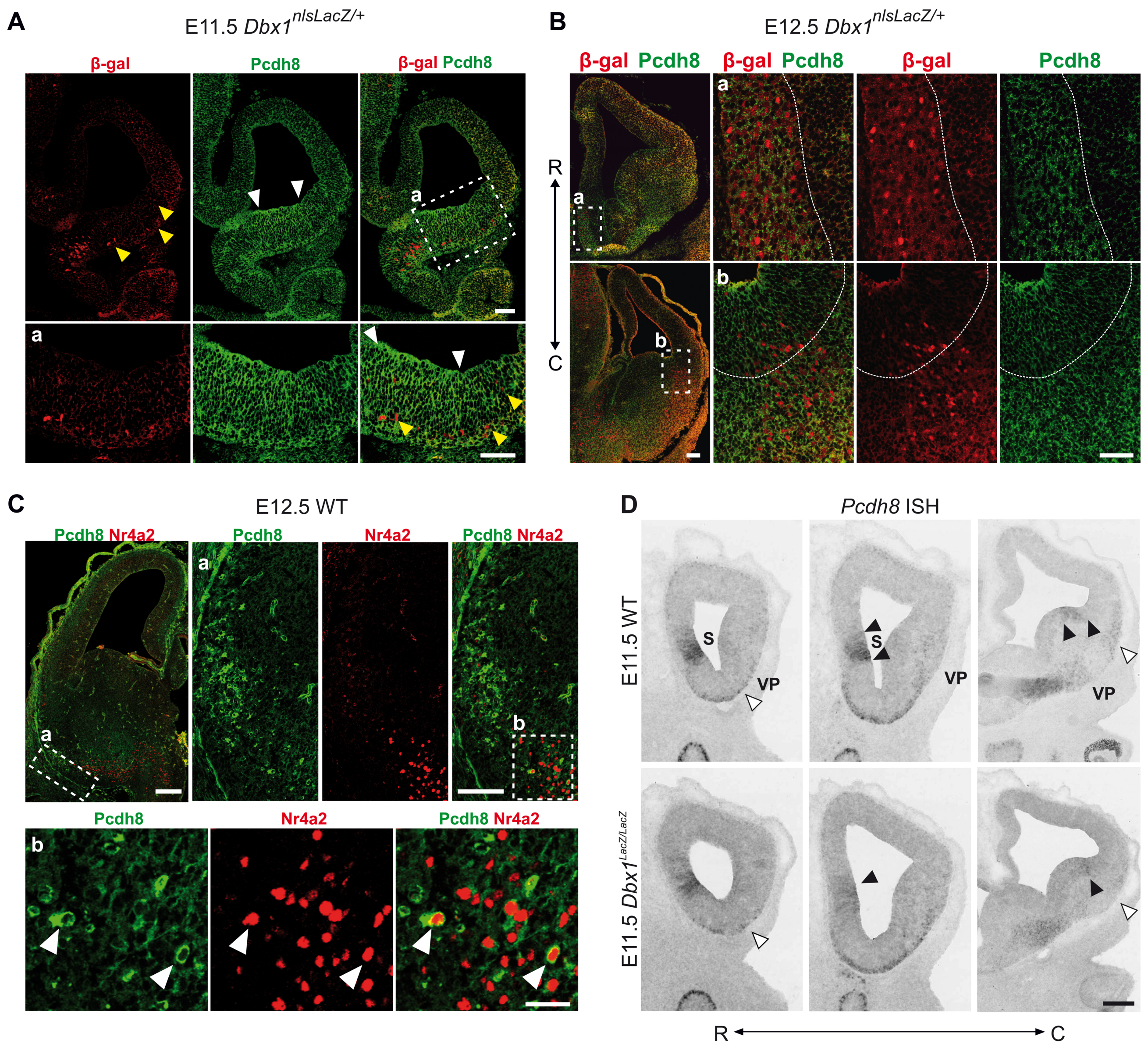

Fig. S5. Pcdh8 and Dbx1 display interrelated expression patterns in the developing telencephalon.

(A, B) Confocal images of β-gal (red) and Pcdh8 (green) co-labeling in coronal brain sections of **(A)** E11.5 and **(B)** E12.5 *Dbx1*$^{nlsLacZ/+}$ embryos. Yellow arrowheads indicate β-gal^{+}Pcdh8^{+} cells, while white arrowheads mark Pcdh8^{+} areas. Dashed boxes (a, b) magnified below the corresponding low-magnification images. In **(B)**, dashed lines outline the borders of stronger Pcdh8 staining and the double arrow line indicates the rostro-caudal (R-C) axis. Scale bars: 200 µm, 100 µm (magnified). **(C)** Confocal images of coronal brain sections from E12.5 WT embryos co-labeled with Pcdh8 (green) and Nr4a2 (red). Dashed boxes (a, b) magnified on the right of the corresponding image. White arrowheads indicate Pcdh8^{+}Nr4a2^{+} cells. Scale bars: 100 µm, 50 µm (a), 25 µm (b). **(D)** Bright-field images of ISH for *Pcdh8* along the rostro-caudal (R-C) axis (double arrow line) in coronal brain sections of E11.5 WT and *Dbx1*$^{LacZ/LacZ}$ (Dbx1 KO) embryos. White and black arrowheads indicate changes of *Pcdh8* expression related to the organization of cells in the ventral postmitotic compartment, and the localization of septum (S) and VP progenitor domains, respectively. Scale bar: 100 µm.

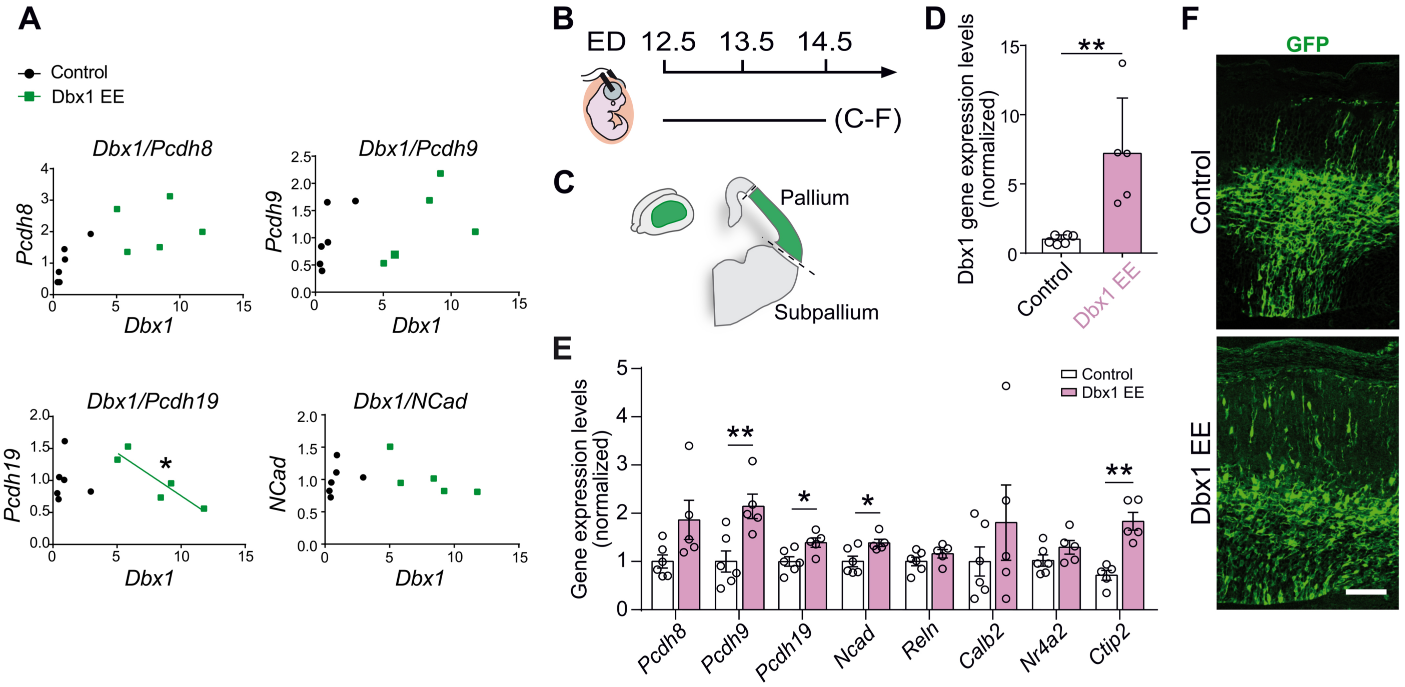

Fig. S6. Dbx1 EE at E12.5 induces expression of different sets of genes in comparison to IUE at E11.5.

(A) qPCR analysis of gene expression upon IUE (E11.5-E13.5) with either a control vector or Dbx1 EE, showing no correlation (*Dbx1/Pcdh8*; *Dbx1/Pcdh9*; *Dbx1/Ncad*) or a negative correlation (*Dbx1/Pcdh19*). **(B)** Timeline of the IUE. ED, electroporation day. **(C)** Electroporated region dissection scheme. **(D, E)** qPCR quantification of the average expression levels of **(D)** *Dbx1* and **(E)** *Pcdh8, Pcdh9, Pcdh19, Ncad, Reln, Calb2, Nr4a2* and *Ctip2* upon IUE (E12.5-E14.5) with either a control vector or Dbx1 EE, normalized internally to *Gapdh* expression. Data are mean ± SEM; circles represent values from independent electroporated embryos (n=6 controls, n=5 Dbx1 EE). Student's t-test: **(D)** **p=0.0014, **(E)** *$p \leq 0.05$; **$p \leq 0.01$. **(F)** Confocal images of GFP (green) in coronal sections of E14.5 mouse brains upon IUE at E12.5 with control vector or Dbx1 EE. Scale bar: 100 µm.

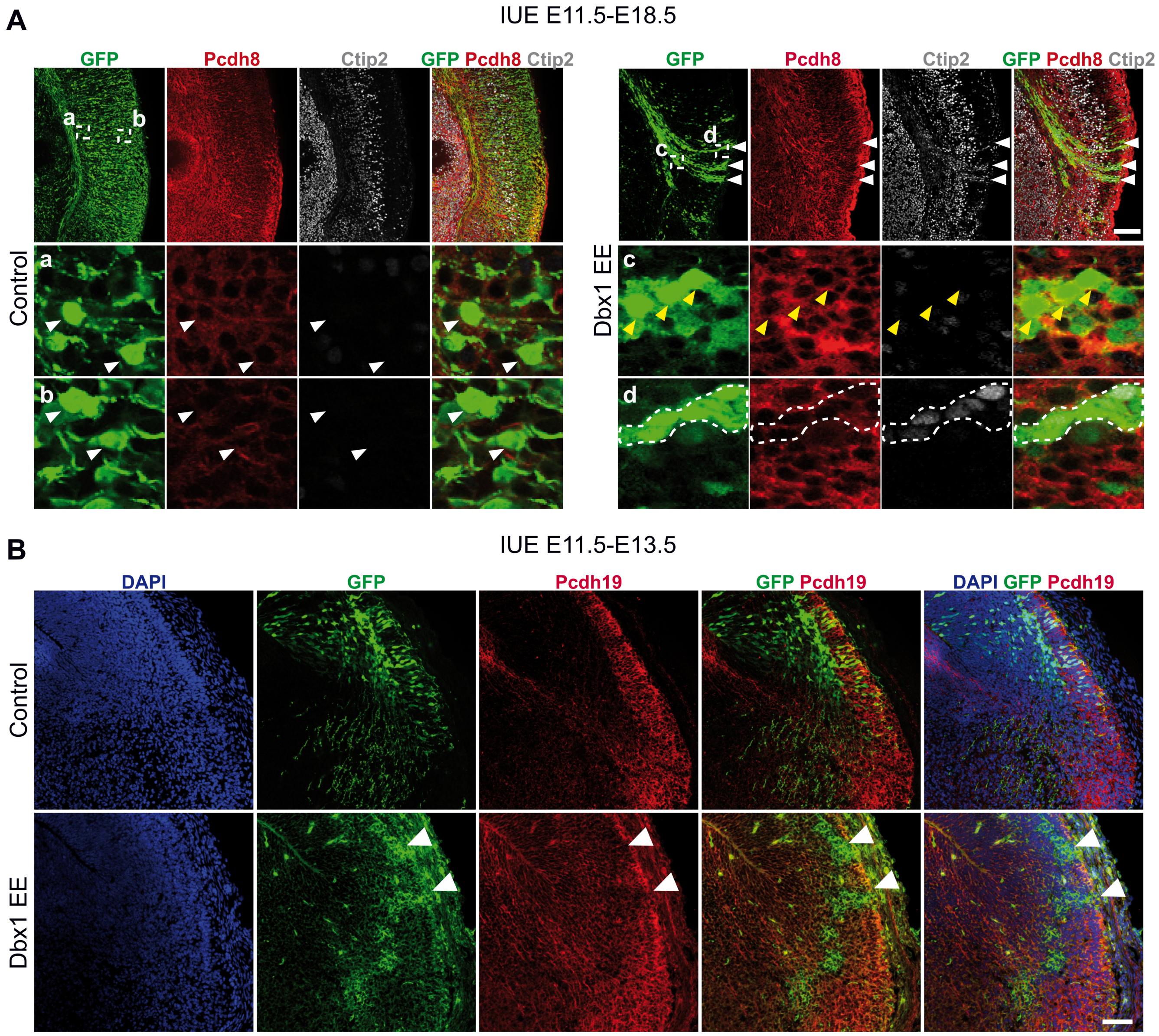

Fig. S7. Dbx1 EE at E11.5 affects cell identity and adhesion.

(A) Confocal images of GFP (green) in coronal sections of E18.5 mouse brain cortices electroporated at E11.5 with control vector or Dbx1 EE, co-labeled for Pcdh8 (red) and Ctip2 (gray). Dashed squares (a, b, c, d) magnified below. White arrowheads indicate $GFP^{+}Pcdh8^{-}Ctip2^{-}$ cells in the control and strongly adherent 'streams' of GFP^{+} cells in Dbx1 EE. Yellow arrowheads in (c) indicate Dbx1 EE $Pcdh8^{high}Ctip2^{-}$ cells below the CP, while the dashed area in (d) shows a group of Dbx1 EE $Pcdh8^{low}Ctip2^{+}$ cells in the CP. Scale bars: 200 µm, 25 µm (magnified). **(B)** Confocal images of GFP (green) in coronal sections of E13.5 mouse brain cortices electroporated at E11.5 with control vector or Dbx1 EE, co-labeled for Pcdh19 (red) with DAPI (blue) counterstaining. White arrowheads indicate $Pcdh19^{-}$ aggregates induced by Dbx1 EE. Scale bar: 100 µm.

Development • Supplementary information

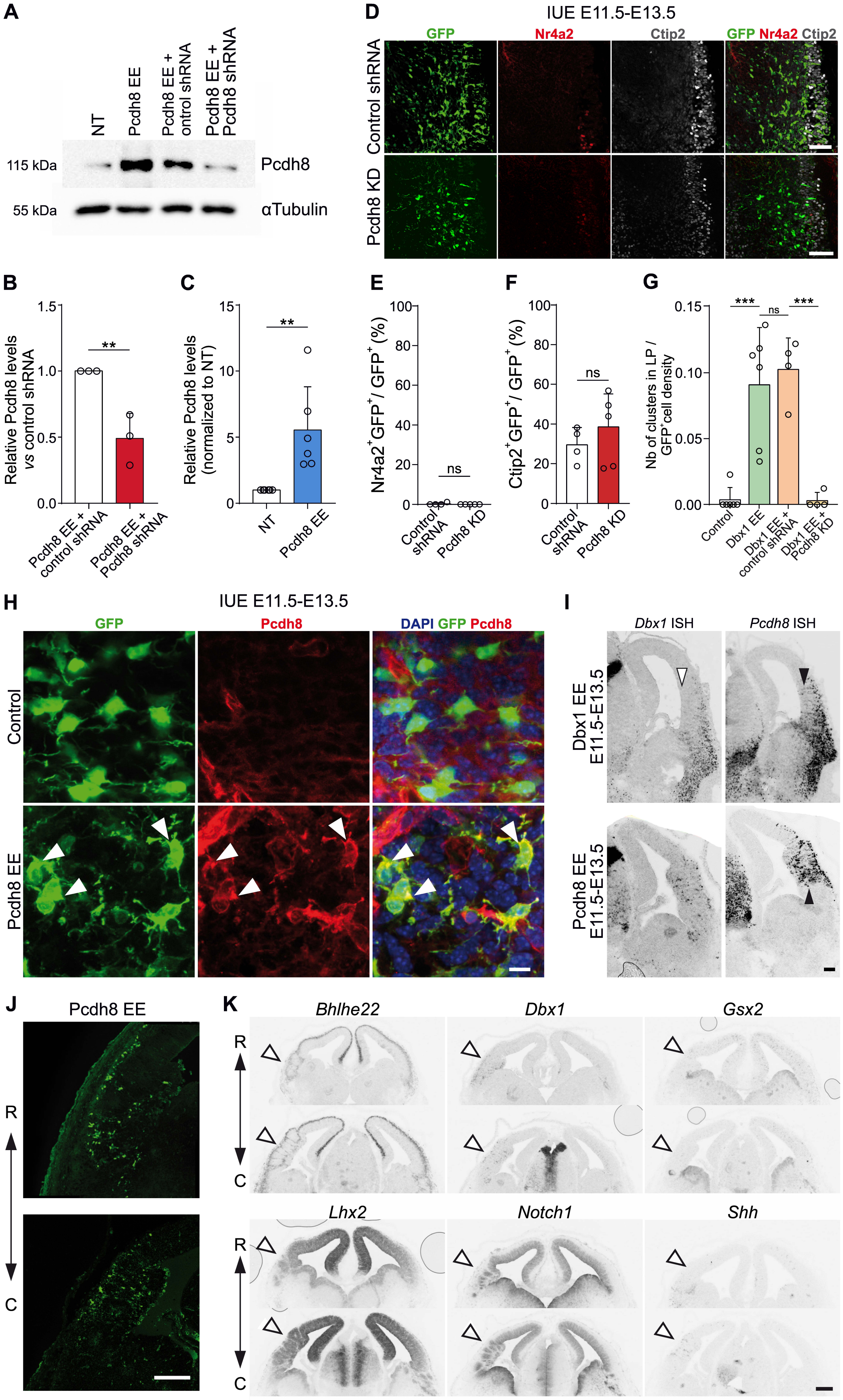
A
NT
Pcdh8 EE
Pcdh8 EE + control shRNA
Pcdh8 EE + Pcdh8 shRNA
115 kDa
Pcdh8
55 kDa
αTubulin
D
IUE E11.5-E13.5
GFP
Nr4a2
Ctip2
GFP Nr4a2 Ctip2
Control shRNA
Pcdh8 KD
B
Relative Pcdh8 levels vs control shRNA
Pcdh8 EE + control shRNA
Pcdh8 EE + Pcdh8 shRNA
C
Relative Pcdh8 levels (normalized to NT)
NT
Pcdh8 EE
E
Nr4a2+GFP+/ GFP+ (%)
Control shRNA
Pcdh8 KD
F
Ctip2+GFP+/ GFP+ (%)
G
Nb of clusters in LP / GFP+cell density
Control
Dbx1 EE
Dbx1 EE + control shRNA
Dbx1 EE + Pcdh8 KD
H
IUE E11.5-E13.5
GFP
Pcdh8
DAPI GFP Pcdh8
Control
Pcdh8 EE
I
Dbx1 ISH
Pcdh8 ISH
Dbx1 EE E11.5-E13.5
Pcdh8 EE E11.5-E13.5
J
Pcdh8 EE
R
C
K
Bhlhe22
Dbx1
Gsx2
Lhx2
Notch1
Shh

Fig. S8. Pcdh8 KD does not affect cell identity but Pcdh8 EE alters the organization of the lateral pallium.

(A) Representative immunoblotting of Pcdh8 protein levels in cell lysates from HEK293T cells 48h post-transfection under the following conditions: non-transfected (NT), Pcdh8 EE, Pcdh8 EE + control shRNA, and Pcdh8 EE + Pcdh8 shRNA. **(B, C)** Densitometric quantification of Pcdh8 signal, normalized to αTubulin, to assess **(B)** the relative percentage of Pcdh8 knockdown (KD) and **(C)** Pcdh8 EE efficacy (n=6 NT and Pcdh8 EE; n=3 Pcdh8 EE + control shRNA and Pcdh8 EE + Pcdh8 shRNA). Data are mean ± SEM; circles represent values from independent transfections. Student's t-test: $^{**}p \leq 0.01$. **(D)** Confocal images of GFP (green) in coronal sections of E13.5 mouse brains electroporated at E11.5 with a control shRNA or Pcdh8-targeted shRNA (Pcdh8 KD), co-labeled with Nr4a2 (red) and Ctip2 (gray). Scale bars: 50 µm. **(E-F)** Percentage of **(E)** $Nr4a2^{+}GFP^{+}$ and **(F)** $Ctip2^{+}GFP^{+}$ cells among GFP^{+} cells. Data are mean ± SEM; circles represent values from independent electroporated embryos (n=4 controls, n=5 Pcdh8 KD). Student's t-test: ns, not significant. **(G)** Quantification of GFP^{+} cluster formation in the lateral pallium. Data are mean ± SEM; circles represent single analysed section values (n=6 controls, n=6 Dbx1 EE, n=4 Dbx1 EE + control shRNA, n=4 Dbx1 EE + Pcdh8 KD) from independent electroporations (from at least 3 animals). One-way ANOVA, *post hoc* Holm-Sidak: ns, not significant; $^{***}p<0.001$. **(H)** Confocal images of GFP (green) in coronal sections of E13.5 mouse brains electroporated at E11.5 with control vector or Pcdh8 EE, co-labeled with Pcdh8 (red) and DAPI (blue) counterstaining. White arrowheads indicate cells overexpressing Pcdh8. Scale bar: 50 µm. **(I)** Bright-field images of ISH for *Dbx1* and *Pcdh8* in coronal sections of E13.5 mouse brains electroporated at E11.5 with Dbx1 EE or Pcdh8 EE. White arrowheads show small number of Dbx1-expressing cells in the VZ, while black arrowheads show cells in the postmitotic compartment. Scale bar: 150 µm. **(J)** Confocal images of GFP (green) along the rostro-caudal (R-C) axis in coronal sections of E13.5 mouse brains electroporated with Pcdh8 EE. Scale bar: 200 µm. **(K)** Bright-field images of ISH for *Bhlhe22*, *Dbx1*, *Gxs2*, *Lhx2*, *Notch1* and *Shh* along the R-C axis in coronal sections of E13.5 mouse brains electroporated at E11.5 with Pcdh8 EE. White arrowheads indicate changes in expression along the DV axis. The caudal images of *Bhlhe22, Dbx1* and *Lhx2* are reproduced from Fig. 5D, as they represent the same experimental condition with bilateral hemispheric representation. Scale bar: 250 µm.

Development • Supplementary information

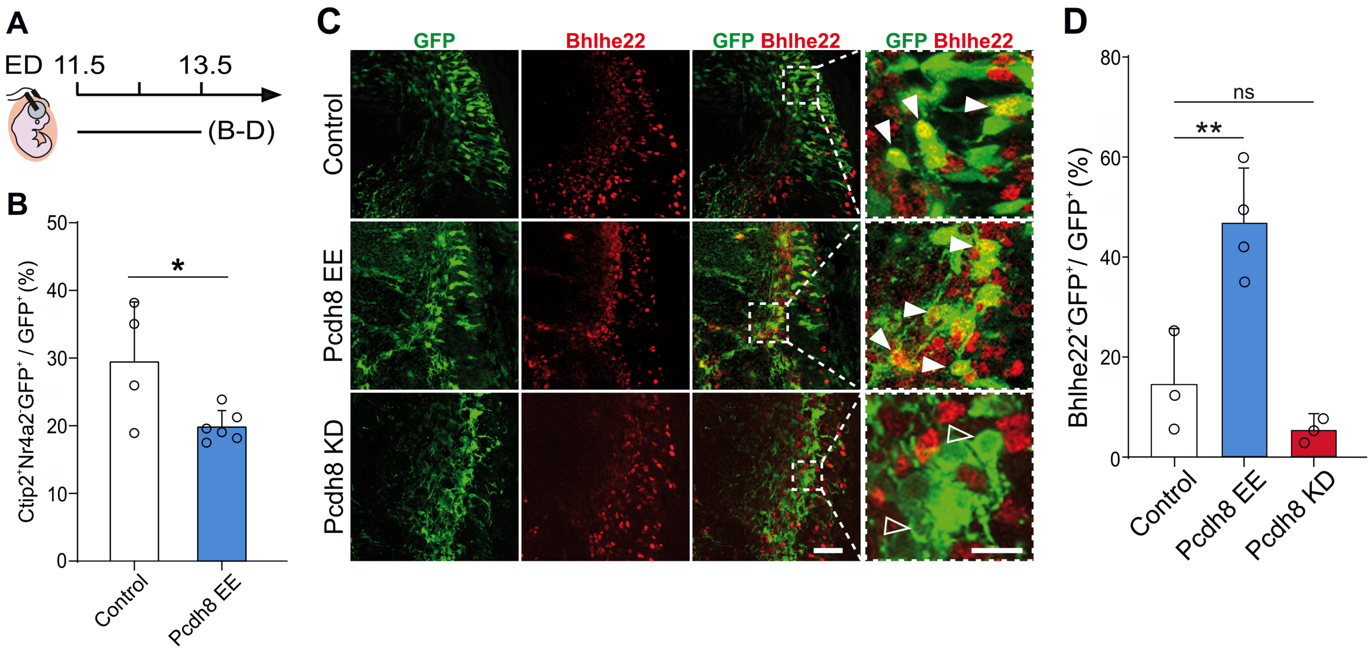

Fig. S9. Pcdh8 EE reduces Ctip2⁺ while promoting Bhlhe22⁺ neurons.

(A) Timeline of the IUE. ED, electroporation day. **(B)** Percentage of Ctip2$^+$Nr4a2$^-$GFP$^+$ cells among GFP$^+$ cells. Data are mean ± SEM; circles represent individual section values (*n*=3 electroporations/condition, 1-2 sections/mouse). Student's *t*-test: **p*=0.0304. **(C)** Confocal images of GFP (green) in coronal sections of E13.5 mouse brains electroporated at E11.5 with control, Pcdh8 EE or Pcdh8 KD (Pcdh8 shRNA), co-labeled with Bhhe22 (red). Dashed squares magnified on the right. White and empty arrowheads indicate GFP$^+$Bhlhe22$^+$ and GFP $^+$Bhlhe22$^-$ cells, respectively. The images depicting the control condition are reproduced from Fig. S2E, which represents the same experimental condition within the same experimental block. Scale bars: 100 µm; 25 µm (magnified). **(D)** Percentage of Bhlhe22$^+$GFP$^+$ cells among GFP$^+$ cells. Data are mean ± SEM; circles represent values from independent electroporated embryos (*n*=3 each condition). One-way ANOVA, *post hoc* Holm-Sidak: ns, not significant, **p*=0.0101, ***p*=0.0027.

Development • Supplementary information

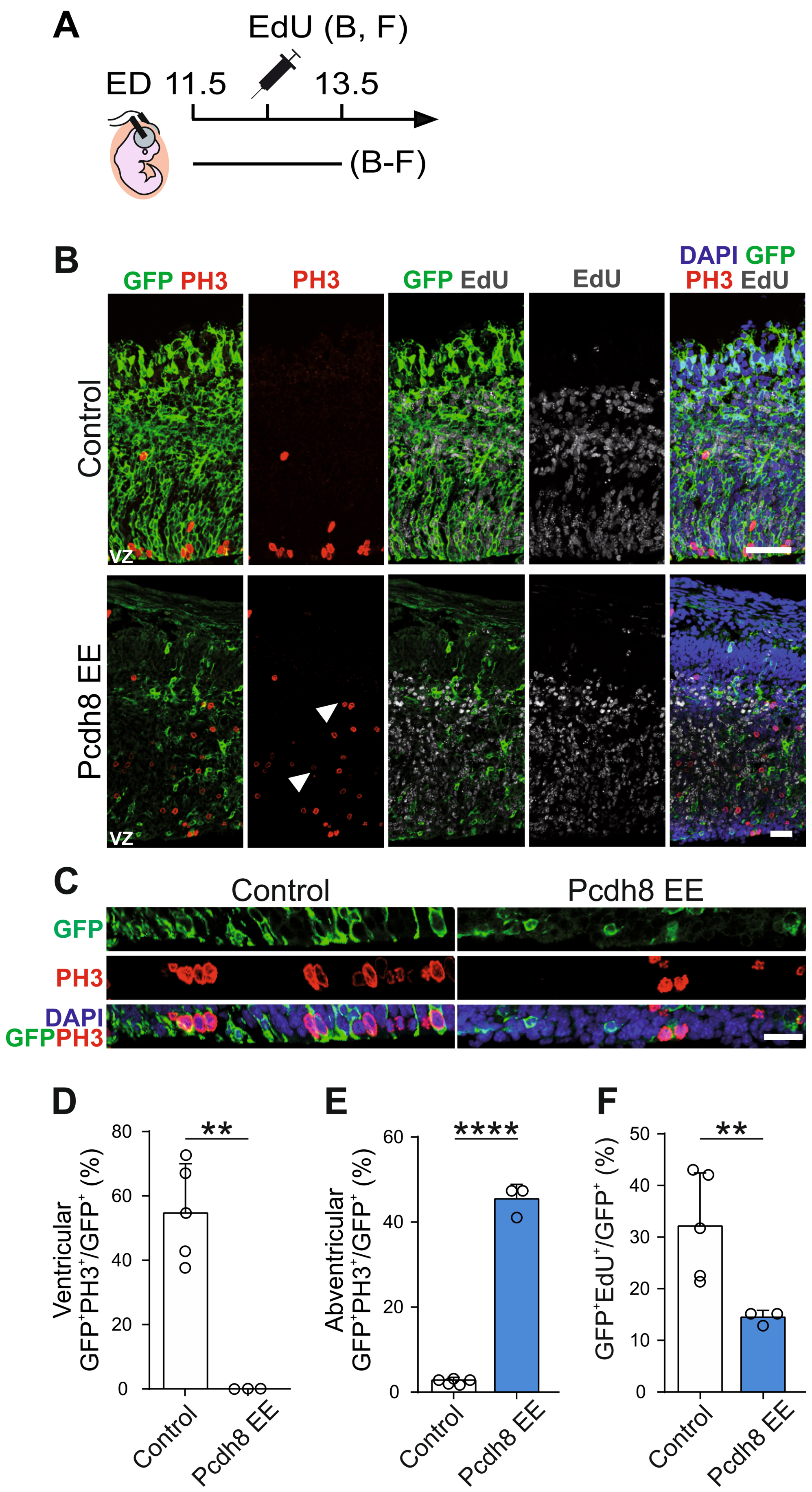

Development • Supplementary information

Fig. S10. Pcdh8 EE alters cell cycle exit.

(A) Schematic timeline of the IUE. ED, electroporation day. **(B)** Confocal images of GFP (green) in coronal sections of E13.5 mouse brains electroporated at E11.5 with control vector or Pcdh8 EE, followed by EdU administration 24h post-electroporation. Sections are co-labeled with PH3 (red), EdU (gray) and DAPI (blue) counterstaining. White arrowheads indicate $PH3^+$ cells outside the VZ. Scale bars: 100 µm. **(C)** Electroporated VZ from **(B)** magnified, showing GFP (green), PH3 (red) and DAPI (blue) labeling. Scale bar: 50 µm. **(D, E)** Percentages of GFP^+PH3^+ cells among GFP^+ cells, aligned either **(D)** with the VZ (ventricular) or **(E)** outside the VZ (abventricular). Data are mean ± SEM; circles represent values from independent electroporated embryos (n=3-5 mice/condition, 2 sections/mouse). Student's t-test: **(D)** **p=0.01, **(E)** ****p<0.001. **(F)** Percentage of GFP^+ EdU^+ cells among GFP^+ cells. Data are mean ± SEM; circles represent values from independent electroporated embryos (n=3-5 mice/condition, 2 sections/mouse). Student's t-test: **p=0.0087.

Table S1. List of reagents used in the study.

REAGENT or RESOURCE	SOURCE	IDENTIFIER
**Antibodies**		
αTubulin (Polyclonal Rabbit)	Invitrogen	PA529444
β-Gal (Polyclonal Chicken)	Abcam	ab9361
Bhlhe22 (Monoclonal Guinea Pig)	Pierani et al., 2001	NA
Ctip2 (Monoclonal Rat)	Abcam	ab18465
Calb2 (Polyclonal Rabbit)	Sawant	7699/3H
DAPI (4', 6-diamidino-2-phenylindole)	Sigma	D9542
Dbx1 (Polyclonal Rabbit)	Pierani et al., 2001	NA
Delta 1(Polyclonal Rabbit)	Santa Cruz	SC-9102
GFP (Polyclonal Chicken)	Aves labs	GFP-1020
Jag1 (Polyclonal Goat)	Santa Cruz	SC-6011
Ncad (Monoclonal Mouse)	BD Bioscience	610921
Nr4a2 (Nurr1) (Polyclonal Goat)	R&D systems	AF2156
Pax6 (Polyclonal Mouse)	Biotech	12323-1-AP
Pcdh8 (Monoclonal Mouse)	Novus Bio	H000051100-M01
Pcdh19 (Polyclonal Rabbit)	Abcam	Ab191198
PH3 (Polyclonal Rabbit)	Millipore	06-570
Reln G10 (Monoclonal Mouse)	Millipore	MAB5364
Tbr2 (Polyclonal Chicken)	Millipore	AB15894
Tle4 (Polyclonal Rabbit)	Abcam	Ab140485
Alexa-488 Donkey Anti-Mouse	Molecular Probes	A-21202
Alexa-488 Donkey Anti-Chicken	Jackson Laboratories	703-545-155
Alexa-555 Goat Anti-Guinea Pig	Molecular Probes	A-21435
Cy3 Donkey Anti-Mouse	Jackson Laboratories	715-165-151
Cy3 Donkey Anti-Rabbit	Jackson Laboratories	711-165-152

Development • Supplementary information

Cy3 Donkey Anti-Goat	Jackson Laboratories	705-165-003
Cyanine 3 Tyramide	Biotechne	6457
Cy5 Donkey Anti-Rat	Jackson Laboratories	712-175-153
Cy5 Donkey Anti-Rabbit	Jackson Laboratories	711-175-152
Cy5 Donkey Anti-Guinea Pig	Jackson Laboratories	706-175-148
Biotin-SP-Conjugated AffiniPure Goat Anti-Mouse	Jackson Laboratories	115-065-146
HRP-conjugated secondary anti-mouse	Jackson Laboratories	115-035-008
HRP-conjugated secondary anti-rabbit	Jackson Laboratories	111-035-008
**Bacterial and Virus Strains**		
One Shot™ TOP10 competent E. coli	Invitrogen	C404010
**Biological Samples**		
**CHEMICALS, PEPTIDES, AND RECOMBINANT PROTEINS**		
**Critical Commercial Assays**		
Alexa Fluor™ 488 Tyramide Super Boost™	Invitrogen	B40912
Anti-DIG-AP	Roche	11093274910
Anti-DIG-POD	Roche	1207733910
Anti-FITC-POD	Roche	1426346910
Click iT EdU Alexa Fluor 647 Imaging Kit	Invitrogen	C10340
DIG-RNA labeling Mix	Roche	11277073910
Fluorescein RNA labeling Mix	Roche	11685619910
GoTag qPCR Master Mix	Promega	A6001
RevertAid First Strand cDNA Synthesis Kit	Thermo Scientific	K1622
Chromium Single Cell 3' Library & Gel Bead Kit	10x Genomics	PN-120267
Neural Tissue Dissociation Kit (P)	Miltenyi Biotec	130-092-628
Mouse Neural Stem Cell Nucleofector® Kit	Lonza	VPG-1004
DNase I, RNase-free	Thermo Scientific	EN0521
**Deposited Data**		
Raw and analyzed single-cell sequencing data	This paper	GEO: GSE229603
R codes for analysis	This paper	[illegible]s://fcauseret.git<br>[illegible].io/septum/
**Experimental Models: Cell Lines**		

Development • Supplementary information

HEK293T	ATCC	CRL-3216
NC929 (L929) cells	(Porlan et al., 2014)	L929
**Experimental Models: Organisms/Strains**		
C57BL/6RJ	Janvier Labs France	SC-C57J-F
Dbx1^{LacZ}	(Pierani et al., 2001)	NA
Rosa^{26YFP}	(Srinivas et al., 2001)	NA
Rosa^{26tdTomato}	(Madisen et al., 2010)	NA
**Oligonucleotides**		
ISH: Bhlhe22-forward TTAGTCGCCTACCTCAACCAA	This paper	NA
ISH: Bhlhe22-reverse TTTCTCCTGGCTCAGAATCAAG	This paper	NA
ISH: Dbx1	Pierani et al., 2001	NA
ISH: Pcdh8-forward AAGAAGGAGCCTTACGGTGC	This paper	NA
ISH: Pcdh8-reverse TGCTACCAGGAGGGGATTCA	This paper	NA
ISH: Pcdh9-forward ACACACCAGACAGTCTCAGC	This paper	NA
ISH: Pcdh9-reverse TGGCCGCCATTGTTGAAAT	This paper	NA
ISH: Pcdh19-forward CACTTGTCTCCTCGGCTGTT	This paper	NA
ISH: Pcdh19-reverse TGAAGGTGGAGCTGCTTTTG	This paper	NA
qPCR:Dbx1-forward CTTGAAGGACTCGCAGGTGA	This paper	NA

Development • Supplementary information

qPCR: Dbx1-reverse<br>TTTGTGGGAAGGGTCTGCTC	This paper	NA
qPCR: Pcdh8-forward<br>ATGTTCGACGTGCTCACCTT	This paper	NA
qPCR: Pcdh8–reverse<br>CTCGAAGTGACAGGCGCTTT	This paper	NA
qPCR: Pcdh9-forward<br>ACCCTTTCCAAACGCTCCTC	This paper	NA
qPCR: Pcdh9-reverse<br>GAGGTCCATCTGTCTGGTGTG	This paper	NA
qPCR: Pcdh19-forward<br>CATCACTTGTCTCCTCGGCT	This paper	NA
qPCR: Pcdh19-reverse<br>TCAGCGATTCTCTTTCCCCTT	This paper	NA
qPCR: Ncad-forward<br>GGGTGTCCAAGGGTGACAAG	This paper	NA
qPCR: Nacd-reverse<br>TTGCTGAATTTCACTTGCAAAGC	This paper	NA
qPCR: Nr4a2-forward<br>GCTCAGCTCGGCGGTC	This paper	NA
qPCR: Nr4a2-reverse<br>CGTCAGATCTCCCTGCCCA	This paper	NA
qPCR: Reln-forward<br>TTACAACGTCCCCCTGGAAG	This paper	NA
qPCR: Reln-reverse<br>TAGGACGACCTCCACATGGT	This paper	NA
qPCR: Calr-forward<br>AGCACTTTGATGCTGACGGA	This paper	NA
qPCR: Calr-reverse<br>TGGACATCATGCCAGAACCC	This paper	NA
qPCR: Ctip2-forward<br>GGAGAACATTGCAGGGCCG	This paper	NA

Development • Supplementary information

qPCR: Ctip2-reverse GGGAAACAGGGTGGGAGAAC	This paper	NA
qPCR: Gapdh-forward: TGACGTGCCGCCTGGAGAAAC	This paper	NA
qPCR: Gapdh-reverse: CCGGCATCGAAGGTGGAAGAG	This paper	NA
**Recombinant DNA**		
pCAGGS-ires-EGFP	Addgene	#32482
psiSTRIKE-CAG-ires-GFP	Gift from Pierre Billuart	Hu et al., 2016
pT3-EFa1-HA-Jag1	Addgene	#46051
Pcdh8 shRNA Top Strand: ACCGGCGTGTGCTAGATGCCAATGACGAAT CATTGGCATCTAGCACACGCCTTTT	This paper	NA
Pcdh8 Scrambled shRNA Top Strand: ACCGGTGGGAATCGCGCTTACAGTACGAAT ACTGTAAGCGCGATTCCCACCTTT	This paper	NA
**Software and Algorithms**		
Adobe Photoshop	Adobe Systems Incorporated	https://www.adobe.com/products/photoshop.html
Excel	Microsoft	Microsoft
Graph Prism	GraphPad Software	https://www.graphpad.com/scientific-software/prism/
Adobe Illustrator	Adobe Systems Incorporated	https://www.adobe.com/products/illustrator.html
Image J	Schneider et al., 2012	https://imagej.nih.gov/ij/
NDP Viewer	Hamamatsu	U12388-01
Image Lab™ software	Biorad	https://www.bio-rad.com/

Development • Supplementary information

**Other**		
NEPA21 electroporator	Nepagene	NEPA21
Amaxa Nucleofector II	Amaxa Biosystems	AAD-1001S
In-Fusion® HD Cloning	Takara Bio	102518
RevertAid First Strand cDNA Synthesis Kit	Thermo Fisher	K1622
Go Tag qPCR Master Mix	Promega	A6001
DAPI (4', 6-diamidino-2-phenylindole)	Invitrogen	D1306
EdU (5-ethynyl-2'-deoxyuridine)	Invitrogen	E10187
B27	Thermo Fisher	12587010
DMEM	Gibco	31966021
DMEM/F-12	Gibco	11320033
Opti-MEM Reduced Serum Medium	Gibco	11058021
Opti-MEM Reduced Serum Medium, Glutamax Supplement	Gibco	51985026
Fetal Bovine Serum (FBS)	Gibco	10270106
Penicillin-Streptomycin (P/S)	Gibco	15140122
Epidermal Growth Factor (EGF)	Gibco	53003018
Fibroblast Growth Factor-basic (FGFb)	Sigma	F0291
Lipofectamine 2000	Invitrogen	11668019
PI cocktail (cOmplete™ tablets)	Roche	4693159001
BCA protein assay reagent kit	Thermo Fisher	23227
TRIzol Reagent	Invitrogen	15596-026
Vilo Kit cDNA synthesis	Invitrogen	11754050
SDS-PAGE on 3-8% tris-acetate gels (NuPAGE, Invitrogen)	Thermo Fisher	EA03785BOX
NuPAGE™ Antioxidant	Invitrogen	NP0005
NuPAGE™ LDS Sample Buffer (4X)	Invitrogen	NP0007
NuPAGE™ Sample Reducing Agent (10X)	Invitrogen	NP0004
NuPAGE™ Tris-Acetate SDS Running Buffer (20X)	Invitrogen	LA0041
NuPAGE™ Transfer Buffer (20X)	Invitrogen	NP00061

Development • Supplementary information

Skimmed milk	Régilait	NA
Nitrocellulose membranes 0.45 (Amersham)	GE Healthcare	GE10600002
Protein standard (HiMark 31-460 kDa)	Invitrogen	LC5699
SuperSignal West Pico Chemiluminescent	Thermo Fisher	34580
Chloroform	VWR	22711.290
Paraformaldehyde (PFA)	VWR	28794.295
Sodium Chloride (NaCl)	VWR	28244.295
NP-40	Sigma	18896
Sodium deoxycholate	Sigma	30970
SDS solution 20%	Sigma	05030-1L-F
Tris-HCl	Sigma	T3253
Tween-20	VWR	28829.296
Horse serum (HS)	Gibco	16050122
In-Fusion HD Cloning Kit	Takara Bio	639650
Tissue-Tek O.C.T compound	Sakura	4583
Vectashield Mounting medium	Vector Labs	H-1000

Development • Supplementary information
